# Factors Associated With Surgical Site Infections After Fasciotomy in Patients With Compartment Syndrome

**DOI:** 10.5435/JAAOSGlobal-D-22-00002

**Published:** 2022-02-21

**Authors:** Nelson Merchan, Bailey Ingalls, Jayden Garcia, John Wixted, Tamara D. Rozental, Carl M. Harper, Arriyan S. Dowlatshahi

**Affiliations:** From the Beth Israel Deaconess Medical Center-Harvard Medical School (Dr. Merchan, Ingalls, and Garcia); the Department of Orthopaedic Trauma, Beth Israel Deaconess Medical Center-Harvard Medical School (Dr. Wixted); the Department of Hand and Upper Extremity Surgery, Beth Israel Deaconess Medical Center, Harvard Medical School (Dr. Rozental, and Dr. Harper); and the Department of Hand and Upper Extremity Surgery, Harvard Medical School (Dr. Dowlatshahi), Boston, MA.

## Abstract

Fasciotomy is the standard of care to treat acute compartment syndrome (ACS). Although fasciotomies often prevent serious complications, postoperative complications can be notable. Surgical site infection (SSI) in these patients is as high as 30%. The objective of this study was to determine factors that increase the risk of SSI in patients with ACS.

**Methods:**

A retrospective review of 142 patients with compartment syndrome over 10 years was done. We collected basic demographics, mechanism of trauma, time to fasciotomy, incidence of SSI, use of prophylactic antibiotics, and type and time to wound closure. Statistical analysis of continuous variables was done using the Student *t*-test, ANOVA, multivariable regression model, and categorical variables were compared using the chi-square test.

**Results:**

Twenty-five patients with ACS (17.6%) developed infection that required additional treatment. In the multivariate regression model, there were significant differences in median time to closure in patients with infection versus those without, odds ratio: 1.06 (Confidence Interval 95% [1.00 to 1.11]), *P* = 0.036. No differences were observed in infection based on the mechanism of injury, wound management modality, or the presence of associated diagnoses.

**Conclusion:**

In patients with ACS, the time to closure after fasciotomy is associated with the incidence of SSI. There seems to be a golden period for closure at 4 to 5 days after fasciotomy. The ability to close is often limited by multiple factors, but the correlation between time to closure and infection in this study suggests that it is worth exploring different closure methods if the wound cannot be closed primarily within the given timeframe.

Acute compartment syndrome (ACS) is a condition wherein the increased intracompartmental pressure leads to inadequate tissue perfusion.^1^ This can lead to irreversible tissue injury and necrosis that may result in a host of outcomes, including functional impairment, loss of limb, and possibly death.^2^ Although a variety of conditions can cause ACS, the most common etiology is trauma.^3,4,5,6^ Timely fasciotomy to surgically release the affected compartments is the standard of care when treating ACS.^7,8^

Once the fasciotomy has been completed, return trips to the operating room are often not a priority, and fasciotomy aftercare is not infrequently passed off to a different surgical service. However, postfasciotomy management should be given due attention because notable complications can still occur, such as surgical site infection (SSI), which can threaten limb and life with a reported incidence up to 30%.^9,10^ The body of literature that explores factors contributing to these high infection rates is lacking.

The primary objective of this study was to identify factors which increase the risk of SSI after fasciotomy in patients with ACS of the upper and lower extremities.

## Methods

We performed a retrospective chart review of 420 patients in one tertiary academic medical center. We extracted the patients from our database using the Current Procedural Terminology code for fasciotomy from July 1, 2009, to July 30, 2019. After manual chart review by three research assistants, 142 patients met the inclusion criteria: fasciotomy for compartment syndrome. We excluded patients who had fasciotomy for conditions other than ACS (i.e., Achilles tendon release and prophylactic fasciotomies) (n = 260). Patients who developed ACS because of infectious process were also excluded (n = 18). We collected basic demographics, Charlson Comorbidity Index (a validated tool that predicts the 10-year mortality in patients with multiple comorbidities), mechanism of trauma, location (upper versus lower extremity), time from injury to fasciotomy, use of perioperative antibiotics, SSI within 1 year, associated diagnoses at the moment of admission [polytrauma and IV drug use (IDU)], results of cultures, time to closure, type of closure (partial versus complete), the use of vacuum-assisted closure system (VAC), and surgical outcomes, such as complete recovery (no loss of function or sensation), nerve damage, or amputation.

Once the entire cohort of 142 patients was identified, we compared the patients who developed SSI with patients who did not develop an infection. We defined SSI as an infection that occurred after fasciotomy within 1 year after the procedure took place. The infection was diagnosed either by the surgeon through clinical criteria, such as positive blood markers (white blood count >10.000/mm^3^/lymphocytes predominant) or by positive cultures.

The data are shown as mean ± SD or median (interquartile range) for continuous variables and compared with the Student *t*-test and ANOVA or Wilcoxon rank sum test for nonparametric data. Data are shown as n (%) for categorical variables and compared with the chi-square test or Fishers exact test. We then performed a multivariable regression model predicting infection in which we followed the “rule of tens” which states that for every 10 events, one independent variable can be included in logistic regression^11,12^ and used two variables for the model: days until closure and perioperative antibiotic. Data from this model are shown as odds ratios and 95% CI. We then compared outcomes of upper and lower extremity compartment syndrome, excluding patients who had concomitant upper and lower extremity involvement. And finally, we compared patients who underwent amputation versus limb salvage. The statistical analysis was completed using SAS 9.4.

## Results

### Patients With and Without Infection

**Figure 1:**
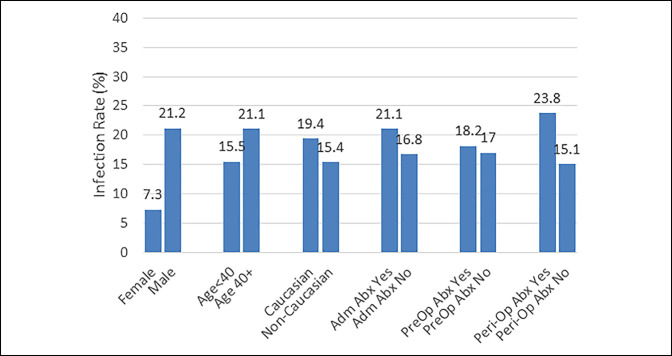
Graph showing rate of infection by demographic characteristics.

Of the 142 patients with confirmed compartment syndrome that were included in this study, 25 (17.6%) went on to develop an infection that required additional medical care, and demographic data are provided in Table 1. Infections were more commonly found in patients who were male (96% of male versus 4% of female patients; *P* ≤ 0.05) (Figure 1). No notable differences in infection rates were noted based on age, body mass index, and Charlson Comorbidity Index (CCI). In addition, no differences were noted in infection rates with the administration of admission or perioperative antibiotics. No differences were observed in infection rates based on the mechanism of injury or the presence of associated diagnoses, including sepsis, polytrauma, and IDU (Table 2). Regarding management, there were no notable differences in infection rates based on the need for débridement, VAC placement, or the type of definitive closure used, including whether the patient had a skin graft or was primarily closed. However, there were significant differences in median time to closure in patients who went on to develop infection versus those who did not (7.0 days versus 4.0; *P* ≤ 0.01) (Table 3).

**Table 1:** Demographics

Factor	Total	Infection	No Infection	*P*
Age (yr)	42 ± 17.3	43.4 ± 15.5	41.7 ± 17.7	0.597
Sex				**0.020**
Female	30	1 (3.3%)	29 (96.7%)	
Male	112	24 (21.4%)	88 (78.6%)	
Body mass index (kg/m^2^)	28.7 ± 6.3	27.5 ± 3.9	28.9 ± 6.7	0.741
Race				0.012
White	90	17	73	
African American	13	0	13	
Asian or Pacific Islander	4	3	1	
Hispanic	13	3	10	
Other	20	2	18	
Charlson Comorbidity Index	1.5 ± 2.3	1.9 ± 2.2	1.4 ± 2.3	0.352

Bold values are statistically significant.

**Table 2:** Antibiotics Use and Mechanism of Injury

Factor	Total	Infection	No Infection	*P*
Admission ABx				0.47
Yes	42	9 (21.4%)	33 (78.6%)	
No	98	16 (16.3%)	82 (83.7%)	
Preoperative ABx				0.825
Yes	96	16 (16.6%)	80 (83.4%)	
No	44	8 (18.0%)	36 (82.0%)	
Perioperative ABx				
Yes	48	12 (25.0%)	36 (75.0%)	0.179
No	84	13 (15.5%)	71 (84.5%)	
Mechanism of injury				0.311
Fracture	58	14 (24.1%)	44 (75.9%)	
Crush	10	1 (10.0%)	9 (90.0%)	
Ischemia/reperfusion	11	3 (27.3%)	8 (72.7%)	
Other (IDU, unknown)	63	7 (11.1%)	56 (88.9%)	

IDU = IV drug use

**Table 3:** Management Postfasciotomy

Factor	Total	Infection	No Infection	*P*
Débridement				0.957
Yes	67	12 (17.9%)	55 (82.1%)	
No	74	13 (17.5%)	61 (82.5%)	
VAC placement?				0.805
Yes	109	19 (17.4%)	90 (82.6%)	
No	31	6 (19.3%)	25 (80.6%)	
Days until closure (Median (IQR))	5.0 (2.0-8.0)	7.0 (3.0-17.0)	4.0 (2.0-7.0)	**0.004 W**
Complete closure				0.438
Yes	124	23 (17.0%)	101 (83.0%)	
Partial closure				0.898
Yes	16	3 (19.0%)	13 (81.0%)	
Graft?				0.771
Yes	38	6 (16.0%)	32 (84.0%)	

Bold values are statistically significant. VAC = Vacuum-assisted Closure system

In the multivariate regression model, the only factor that was independently associated with infection was again time to closure: OR: 1.06 (95% CI, 1.00 to 1.11), *P* = 0.036, whereas the use of perioperative antibiotics was not: odds ratio 0.53 (CI, 0.21 to 1.32), *P* = 0.172.

### Upper Versus Lower Extremity ACS

Although there was no significant difference in the rate of infection between the upper and lower extremities, 7.1% versus 19.6%, *P* = 0.117, the results of a chi-square analysis demonstrate a significant association between the mechanism of injury and the location of compartment syndrome, with most of the LE cases being the result of a fracture (51.4%), whereas most of the UE cases were by IDU or unknown etiologies (53%) and ischemia/reperfusion (25%) *P* ≤ 0.001. There also seemed to be a significant difference in their management, including significant association between extremity location and the use of admission antibiotics (50% of UE cases received admission antibiotics versus 22.9% of LE cases; *P* = 0.004), débridement during surgery (63% of UE cases débrided versus 43.9% of LE cases; *P* = 0.076), and the use of VAC (57.7% of UE cases versus 83.2% of LE cases; *P* = 0.004). Looking at patient outcomes, there was a significant association between UE ACS and nerve damage, 32.1% versus 15% of LE cases; *P* = 0.037. Both the UE and LE groups were comparable for time to closure, skin graft use, associated diagnoses, and amputation. None of the patients with fracture underwent implant removal, and only four patients presented with open fractures. All these results are summarized in the Table 4.

**Table 4:** Location

Factor	n = 135	Upper Extremity, n = 28	Lower Extremity, n = 107	*P*
Infection				0.117
Yes	23	2 (8.7%)	21 (91.3%)	
No	112	26 (23.2%)	86 (76.8%)	
Age	42.3 ± 17.5	49.2 ± 19.4	40.4 ± 16.6	0.017
BMI	28.7 ± 6.4	29.0 ± 9.6	28.6 ± 5.2	0.829
Sex				0.256
Female	30	4 (13.3%)	26 (86.7%)	
Male	105	24 (22.9%)	81 (77.1%)	
Race				0.402
White	87 (65.4%)	15 (17.2%)	72 (88.8%)	
African American	12 (9.0%)	3 (25.0%)	9 (75.0%)	
Asian or Pacific Islander	3 (2.3%)	0 (0.0%)	3 (100%)	
Hispanic	12 (9.0%)	4 (33.3%)	8 (66.7%)	
Other	19 (14.3%)	6 (31.5%)	13 (68.5%)	
ASA	2.2 ± 1.0	3.1 ± 1.0	2.0 ± 0.9	**<0.001**
Charlson Comorbidity Index	1.5 ± 2.3	2.1 ± 2.6	1.3 ± 2.2	0.124
Mechanism of injury				**<0.001**
Fracture	57 (42.2%)	2 (3.5%)	55 (96.5%)	
Crush	10 (7.4%)	4 (40.0%)	6 (60.0%)	
Ischemia/reperfusion	10 (7.4%)	7 (70.0%)	3 (30.0%)	
Other (IDU, unknown)	58 (43.0%)	15 (25.8%)	43 (74.2%)	
Admission ABx				**0.004**
Yes	38	14 (36.8%)	24 (63.2%)	
Preoperative ABx				0.575
Yes	92	18 (19.5%)	74 (80.5%)	
Perioperative ABx				0.176
Yes	47	13 (27.6%)	34 (72.4%)	
Isolated injury?				0.988
Yes	93	20 (21.5%)	73 (78.5%)	
Débridement				0.076
Yes	64	17 (26.5%)	47 (73.5%)	
VAC placement?				**0.004**
Yes	104	15 (14.4%)	89 (85.6%)	
Days until closure (Median [IQR])	5.0 (2.0-8.0)	3.0 (0.0-7.0)	5.0 (2.0-8.0)	0.233 W
Complete closure				**0.015**
Yes	119	21 (17.6%)	98 (82.4%)	
Partial closure				**0.008**
Yes	15	7 (46.6%)	8 (53.4%)	
Graft?				0.208
Yes	38	10 (26.3%)	28 (73.7%)	
Complete recovery				0.473
Yes	66	12 (18.2%)	54 (81.8%)	
Amputation				0.436
Yes	6	2 (33.3%)	4 (66.7%)	
Nerve damage				0.037
Yes	25	9 (36.0%)	16 (64.0%)	

Bold values are statistically significant. IDU = IV drug use, VAC = Vacuum-assisted Closure system

### Salvage Versus Amputation

Cases were also assessed based on whether they ultimately resulted in limb amputation (Table 5). A significant association was observed between postfasciotomy infection and amputation (16% of cases with infection resulted in amputation versus 2.6% without infection; *P* = 0.004). The results also demonstrated an association between mechanism of injury and amputation, with amputation occurring more commonly in ACS secondary to ischemia (36.4% *P* ≤ 0.001). In addition, ACS because of an isolated injury was less likely to result in amputation as opposed with associated conditions, such as polytrauma (2.1% versus 12.2%, *P* = 0.007). Patients who presented with ACS in the setting of IDU had a significantly higher rate of amputation (50% with associated IDU resulted in amputation versus 3.2% without; *P* ≤ 0.001). Cases requiring amputation also had a significantly longer median time to closure (21.5 days versus 5.0; *P* = 0.001). No notable associations were observed between amputation and VAC placement and the type of definitive closure.

**Table 5:** Amputation

Factor	Amputation, N = 7	No Amputation, N = 135	*P*
Infection			**0.004**
Yes	4 (57.1%)	21 (15.5%)	
No	3 (42.9%)	114 (84.5%)	
Mechanism of injury			**<0.001**
Fracture	2	56	
Crush	0	10	
Ischemia/reperfusion	4	7	
Other (IDU, unknown)	1	62	

Bold values are statistically significant. IDU = IV drug use

### Microbiology

We also evaluated the results of the cultures from the infected cohort. Cultures were taken in 20 of the 25 patients with infection, of which all were positive. The most common wound isolates were coagulase-positive *Staphylococcus* (n = 10), coagulase-negative *Staphylococcus* (n = 4) and *Streptococcus* (n = 5) cultures. Less frequent microorganisms were *Bacillus* not anthracis (n = 1), *Enterobacter cloacae* (n = 2), *Mycoplasma* (n = 1), *Pseudomonas aeruginosa* (n = 1), *Acinetobacter* (n = 1), *Enterococcus* (n = 3), and *Corynebacterium* (n = 1). Eight patients (27.5%) had mixed bacterial flora.

## Discussion

ACS carries a substantial risk of morbidity and mortality, including chronic pain, permanent functional impairment, amputation, and death.^2^ Timely surgical intervention with fasciotomies to release the increased intracompartmental pressure is the standard of care but does not guarantee a fully functional limb. A problematic complication is the development of SSI, which itself can lead to sepsis, amputation, and death. Previous studies have demonstrated that the rate of postoperative infection in cases of ACS treated with decompressive fasciotomy is as high as 30%.^10^ Yet, there is a lack of studies examining the factors that may be contributing to this high infection rate.

### Patient Factors

In our study, patient characteristics, such as age, did not play a role in the rate of infection as in contrast with other studies that increasing age was an independent factor in SSI posterior to a fasciotomy.^13^ For sex, men were more likely than women to develop a postoperative infection; this could be possible due to unmeasurable confounders like the setting of the mechanism of trauma. Other patient factors, such as body mass index and CCI, were not found to be associated with infection rates, suggesting that time to fasciotomy and other factors surrounding treatment may be more important than the patients' baseline health status on admission. Furthermore, mechanism of injury, location of injury (UE versus LE), and associated injuries at the time of admission (sepsis, polytrauma, and IDU overdose) were not associated with a change in the risk for infection. Even in patients who presented with compartment syndrome due to IDU, there was no notable difference. Given the differences in etiologies play a major role which likely eclipses the time to closure or treatment, we believe that the fact that there were no notable differences is an interesting finding and warrants additional research. This again supports the idea that the initial management of ACS at the time of admission has a larger effect on the outcome than how the patient presented and how the injury occurred.

### Time to Closure

To further support the importance of treatment decisions, a factor independently associated with the likelihood of infection was time to closure after fasciotomy. Patients who developed infections had a median of 7 days until closure, whereas patients who did not develop an infection had a median of 4 days from fasciotomy to closure. The ability to close is often limited by swelling within the compartments, but the strong correlation between time to closure and infection risk could suggest that it is worth exploring other methods if the wound cannot be closed primarily within the given time frame. Other factors, such as débridement at the time of surgery and the type of closure, were not associated with a change in the risk for infection. This serves us to emphasize that with ACS, when considering the risk of infection, the key factor is not necessarily the type of treatment, but the timing. In our center, these patients were receiving antibiotic therapy postoperatively for at least 5 days for those with positive cultures or prophylactically when high suspicion for infection existed.

The time to closure and its correlation with infection have been previously studied. Crowley et al^14^ performed a literature review to determine the effect of the time on infection rates after open fractures. They reviewed multiple studies regarding the timing of closure when evaluating “immediate, early, and delayed closure,” and they conclude that early closure of open fractures is recommended to decrease the rate of infections.

In the study presented by Hake et al,^10^ they conclude that the use of VAC played an important role in developing infection in the surgical site posterior to a fasciotomy. This is an interesting finding because of the well-known effect of VAC on the management of complicated wounds.^13,15,16^ In our experience, 18% of the patients with VAC developed infection versus 81.2% who did not, although this was not notable.

### Conventional Use of Prophylactic Antibiotics

One factor that was not found to have a strong association with infection rates was the use of admission or perioperative antibiotics at the time of fasciotomy. Although this may seem counterintuitive, it is consistent with our observation that patient characteristics and factors surrounding the patient's initial presentation (mechanism of injury) were not found to be associated with a higher infection rate. In analogy, the administration of a single perioperative antibiotic dose at the time of fasciotomy does not change the patient's odds of developing infection. Rather, the infection risk is most strongly associated with the amount of time the fasciotomy is left open. Thus, in our sample, the postoperative infections are likely stemming from bacterial wound colonization in the postfasciotomy period. This could suggest that prophylactic antibiotics may be more useful postoperatively if a wound cannot be closed within a given period; however, more research with larger sample sizes is needed to draw more definitive conclusions. Based on our data, it would be compelling to obtain wound cultures on postfasciotomy day 4 if the wounds cannot be closed by then and to start prophylactic antibiotics at that time and adjust them according to culture results. The suggested empiric coverage would consist of intravenous vancomycin and cefepime, to be adjusted when culture data result.

We also examined differences in upper and lower extremity ACS outcomes. Notable differences were observed in the etiology of UE versus LE ACS, with fractures more common in LE ACS and ischemia/infection more common in UE ACS. Notable differences were also observed in many aspects of management, including the use of admission antibiotics, VAC placement, débridement, and the type of definitive closure. However, despite these notable differences in mechanism of injury and management, there were no differences in infection rates. In addition, there was no notable difference in median days to closure. This again underscores the importance of time to closure, even in the face of many other differences in ACS management.

Velmahos et al^9^ discussed the importance of the location of the injury in the setting of infections in compartment syndrome. They conclude that better blood supply and technically easier repairs in the upper extremities might be reasons for better outcomes compared with the lower extremity fasciotomy.

Although not the primary outcome measure, we also examined which factors were associated with amputation. Median time to closure was again markedly associated with rates of amputation, emphasizing the importance of this factor in ACS outcomes. Of note, other factors that were associated with eventual amputation included mechanism of injury and associated diagnoses, both of which were not associated with the risk for postoperative infections. Patients who developed ACS secondary to ischemia had a markedly higher risk of amputation than all other causes of ACS (36.4%). This has been recently explained by Rothenberg et al where they concluded that in the setting of acute limb ischemia and posterior revascularization, delayed fasciotomy was associated with an increase in risk of major amputation at 30 days.^17^

In addition, patients who had the associated diagnosis of IDU were markedly more likely to eventually undergo amputation. Although an uncommon complication, previous studies have reported amputation in patients with complicated IDU.^18,19^ Patients who went on to develop postoperative infections were also more likely to undergo amputation (13.1% of cases with infection resulted in amputation versus 3.1% without infection), emphasizing the importance of infection prevention.

This study has its limitations. This was a retrospective database study at a single institution. The power of this study is also limited by the relatively small number of postoperative infections. In addition, we tried to maintain our patient population the more homogenous as possible by adequately grouping the patients by their cause of ACS; however, the severity and categorization of the injuries were also an important limitation of this study. Although in all cases, the patients were diagnosed presenting with ACS, not all the patients underwent measurement of compartments to have a more objective diagnosis.^20,21,22,23^ In an effort to elucidate whether patients with ACS due to infections were a cofounder, we performed all the analyses with and without this subgroup. Again, this did not change the outcomes of this study.

In conclusion, prehospital factors, including a patient's baseline health status and mechanism of injury, were not associated with the risk of postfasciotomy infection in patients with ACS in our study, whereas the time to fasciotomy closure is strongly correlated with infection rates. In our cohort, we identified a golden period of closure at 4 to 5 days after fasciotomy. Additional studies using larger sample sizes are warranted, given the interesting conclusions of this study. Future studies could also shed light on the relationship between factors, such as sex and infection rates, and whether there is an intrinsic difference in wound healing leading to different outcomes or confounding factors relating to injury or management decisions. A better understanding of the factors associated with postoperative infections in ACS will help to both identify patients most at risk and help shape data-driven protocols aimed at improving overall outcomes.

## References

[1] Matsen FA III: Compartmental syndrome. An unified concept. Clin Orthop Relat Res 1975;113:8-14. 1192678

[2] von Keudell AG Weaver MJ Appleton PT : Diagnosis and treatment of acute extremity compartment syndrome. Lancet 2015;386:1299-1310. 26460664 10.1016/S0140-6736(15)00277-9

[3] McQueen MM Gaston P Court-Brown CM: Acute compartment syndrome. Who is at risk? J Bone Joint Surg Br 2000;82:200-203. 10755426

[4] Chim H Soltanian H: Spontaneous compartment syndrome of the forearm in association with nephrotic syndrome and transient bacteremia. J Surg Case Rep 2012;2012:11. 10.1093/jscr/2012.8.11 PMC3649580 24960769

[5] Ridha A Khan A Al-Abayechi S Puthenveetil V: Acute compartment syndrome secondary to rhabdomyolysis in a sickle cell trait patient. Lancet 2014;384:2172. 25497201 10.1016/S0140-6736(14)61944-9

[6] Wang KL Li SY Chuang CL Chen TW Chen JY: Subfascial hematoma progressed to arm compartment syndrome due to a nontransposed brachiobasilic fistula. Am J Kidney Dis 2006;48:990-992. 17162155 10.1053/j.ajkd.2006.08.020

[7] Jensen SL Sandermann J: Compartment syndrome and fasciotomy in vascular surgery. A review of 57 cases. Eur J Vasc Endovasc Surg 1997;13:48-53. 9046914 10.1016/s1078-5884(97)80050-0

[8] Rorabeck CH: The treatment of compartment syndromes of the leg. J Bone Joint Surg Br 1984;66:93-97. 6693486 10.1302/0301-620X.66B1.6693486

[9] Velmahos GC Theodorou D Demetriades D : Complications and nonclosure rates of fasciotomy for trauma and related risk factors. World J Surg 1997;21:247-253; discussion 253. 9015166 10.1007/s002689900224

[10] Hake ME Etscheidt J Chadayammuri VP Kirsch JM Mauffrey C: Age and dressing type as independent predictors of post-operative infection in patients with acute compartment syndrome of the lower leg. Int Orthop 2017;41:2591-2596. 28730321 10.1007/s00264-017-3576-1

[11] Peduzzi P Concato J Kemper E Holford TR Feinstein AR: A simulation study of the number of events per variable in logistic regression analysis. J Clin Epidemiol 1996;49:1373-1379. 8970487 10.1016/s0895-4356(96)00236-3

[12] Vittinghoff E McCulloch CE: Relaxing the rule of ten events per variable in logistic and Cox regression. Am J Epidemiol 2007;165:710-718. 17182981 10.1093/aje/kwk052

[13] Novak A Khan WS Plamer J: The evidence-based principles of negative pressure wound therapy in trauma & orthopedics. Open Orthop J 2014;8:168-177. 25067971 10.2174/1874325001408010168 PMC4110388

[14] Crowley DJ Kanakaris NK Giannoudis PV: Debridement and wound closure of open fractures: The impact of the time factor on infection rates. Injury 2007;38:879-889. 17532320 10.1016/j.injury.2007.01.012

[15] Zannis J Angobaldo J Marks M : Comparison of fasciotomy wound closures using traditional dressing changes and the vacuum-assisted closure device. Ann Plast Surg 2009;62:407-409. 19325346 10.1097/SAP.0b013e3181881b29

[16] Kakagia D Karadimas EJ Drosos G Ververidis A Trypsiannis G Verettas D: Wound closure of leg fasciotomy: Comparison of vacuum-assisted closure versus shoelace technique. A randomised study. Injury 2014;45:890-893. 22377275 10.1016/j.injury.2012.02.002

[17] Rothenberg KA George EL Trickey AW Chandra V Stern JR: Delayed fasciotomy is associated with higher risk of major amputation in patients with acute limb ischemia. Ann Vasc Surg 2019;59:195-201. 31034949 10.1016/j.avsg.2019.01.028

[18] Ilic A Stevanovic K Pejkic S : Vascular injuries in intravenous drug addicts-a single-center experience. Ann Vasc Surg 2020;67:185-191. 32335251 10.1016/j.avsg.2020.02.041

[19] Mittapalli D Velineni R Rae N Howd A Suttie SA: Necrotizing soft tissue infections in intravenous drug users: A vascular surgical emergency. Eur J Vasc Endovasc Surg 2015;49:593-599. 25805328 10.1016/j.ejvs.2015.02.002

[20] McQueen MM Court-Brown CM: Compartment monitoring in tibial fractures. The pressure threshold for decompression. J Bone Joint Surg Br 1996;78:99-104. 8898137

[21] Shadgan B Menon M O'Brien PJ Reid WD: Diagnostic techniques in acute compartment syndrome of the leg. J Orthop Trauma 2008;22:581-587. 18758292 10.1097/BOT.0b013e318183136d

[22] Hammerberg EM Whitesides TE Jr Seiler JG III: The reliability of measurement of tissue pressure in compartment syndrome. J Orthop Trauma 2012;26:24-32; discussion 32. 21918480 10.1097/BOT.0b013e31822908cf

[23] Wong JC Vosbikian MM Dwyer JM Ilyas AM: Accuracy of measurement of hand compartment pressures: A cadaveric study. J Hand Surg Am 2015;40:701-706. 25648783 10.1016/j.jhsa.2014.12.003

